# Características sociodemográficas y clínicas de la sífilis gestacional en Cali, 2018

**DOI:** 10.7705/biomedica.6003

**Published:** 2021-10-15

**Authors:** Juliana Benítez, María Alejandra Yépez, Mauricio Hernández-Carrillo, Diana Milena Martínez, Ángela Cubides-Munévar, Jorge Alirio Holguín-Ruiz, Martín Alonso Muñoz

**Affiliations:** 1 Programa de Ginecología y Obstetricia, Universidad Libre, Cali, Colombia Universidad Libre Programa de Ginecología y Obstetricia Universidad Libre Cali Colombia; 2 Posgrado en Ciencias Biomédicas y Psiquiatría, Universidad del Valle, Cali, Colombia Universidad del Valle Posgrado en Ciencias Biomédicas y Psiquiatría Universidad del Valle Cali Colombia; 3 Facultad de Ciencias de la Salud, Universidad Libre, Cali, Colombia Universidad Libre Facultad de Ciencias de la Salud Universidad Libre Cali Colombia; 4 Grupo de Investigación en Salud Pública, Fundación Universitaria San Martín, Cali, Colombia Universidad San Martín Grupo de Investigación en Salud Pública Fundación Universitaria San Martín Cali Colombia; 5 Departamento de Salud Pública y Epidemiología, Pontificia Universidad Javeriana, Cali, Colombia Pontificia Universidad Javeriana Departamento de Salud Pública y Epidemiología Pontificia Universidad Javeriana Cali Colombia; 6 Área de Vigilancia Epidemiológica, Secretaría de Salud Municipal de Cali, Cali, Colombia Área de Vigilancia Epidemiológica Secretaría de Salud Municipal de Cali Cali Colombia; 7 Área de Vigilancia Epidemiológica, Secretaría de Salud Municipal de Cali, Cali, Colombia Área de Vigilancia Epidemiológica Secretaría de Salud Municipal de Cali Cali Colombia

**Keywords:** sífilis congénita, sífilis latente, serodiagnóstico de la sífilis, sífilis/epidemiología, prevalencia, salud pública, Syphilis, congenital, syphilis, latent, syphilis serodiagnosis, syphilis/ epidemiology, prevalence, public health

## Abstract

**Introducción.:**

La sífilis gestacional se considera de interés en salud pública por las repercusiones que tiene en la madre y el hijo. Pese a tener protocolos para su notificación, diagnóstico y manejo, en Colombia se siguen evidenciando limitaciones en su control.

**Objetivo.:**

Describir las características sociodemográficas, clínicas y de distribución espacial de las pacientes con sífilis gestacional en Cali, Colombia, en el 2018.

**Materiales y métodos.:**

Se hizo un estudio transversal con 427 casos de sífilis gestacional reportados al Sistema de Vigilancia en Salud Pública (Sivigila). Para el procesamiento estadístico, se utilizó el programa R, versión 3.5.3. Las variables cualitativas se presentan como proporciones y, las cuantitativas, mediante medidas de tendencia central y dispersión, Para la distribución espacial, se usó el programa Qgis 3.0.

**Resultados.:**

La razón de sífilis gestacional fue de 17 casos por 1.000 vivos (incluidos los mortinatos). El 57,1 % de las pacientes pertenecía al régimen subsidiado de salud y el 16,6 % no estaba asegurado. El 90,4 % de los casos se diagnosticó durante el embarazo; el 47,2% recibió tres dosis de penicilina y el 57,6 % de los contactos recibió tratamiento.

**Conclusiones.:**

La tasa de sífilis gestacional en Cali en el 2018 fue superior a la nacional y la enfermedad se presentó con mayor frecuencia en mujeres gestantes en condición de vulnerabilidad socioeconómica, lo que coincidió con la distribución espacial en general. Se evidenció la falta de oportunidad en la detección temprana y el manejo de la infección tanto en las pacientes como en sus contactos, lo cual dificulta el control de la enfermedad y refleja la inadecuada aplicación de la ruta integral de atención en salud materno-perinatal.

## Abstract (en)

**Introduction:**

Gestational syphilis is considered an event of public health interest given its impact on mother and child. In Colombia, despite having specific protocols for its notification, diagnosis, and management, there are still limitations in its control.

**Objectives:**

To describe the sociodemographic and clinical characteristics, as well as the spatial distribution of gestational syphilis in Cali, Colombia, in 2018.

**Materials and methods:**

We conducted a cross-sectional study of 427 gestational syphilis cases reported to the Colombian national epidemiological surveillance system (Sivigila). For the statistical processing, we used the R program, version 3.5.3. We expressed qualitative variables as proportions and quantitative ones through central tendency and dispersion measures, and to establish the spatial distribution we used the Qgis program, version 3.0.

**Results:**

The prevalence of gestational syphilis was 17 cases per 1,000 live births (including stillbirths); 57.1% of patients belonged to the subsidized healthcare system and 16.6% had no health insurance; 90.4% of cases were diagnosed during pregnancy; 47.2% of the pregnant women received three doses of penicillin, yet only 57.6% of contacts were treated.

**Conclusions:**

The prevalence of gestational syphilis in Cali during 2018 exceeded the national rate with a higher frequency among women in socioeconomic vulnerability conditions consistent with the general spatial distribution. There was a lack of opportunity in the early detection and management of the infection both among pregnant women and their contacts, which hinders the control of the disease and reflects the inadequate application of the comprehensive maternal and perinatal health care route guidelines.

La sífilis es una infección de transmisión sexual exclusiva del ser humano, producida por la bacteria *Treponema pallidum*, de la familia de las espiroquetas, la cual penetra el organismo a través de la piel o mucosas lesionadas. Representa un grave problema de salud pública, con una carga que supera los 12 millones de individuos a nivel global ^1^^,^^2^. Se calcula que alrededor de 1 a 2 millones de mujeres gestantes se infectan anualmente a nivel mundial y, entre 692.000 y 1’530.000, no reciben tratamiento ^1^^,^^3^.

La sífilis se conoce como la “gran simuladora” debido a la falta de especificidad y variedad de sus manifestaciones clínicas y se considera una infección sistémica crónica ^4^^-^^6^. Se transmite por vía sexual y de manera vertical a través de la placenta durante el embarazo, o por contacto del feto con lesiones activas de la madre en el canal del parto ^6^.

Las manifestaciones aparecen con la sífilis primaria; el 50 % de las pacientes cursan con chancro sifilítico en el sitio de inoculación, el cual se resuelve espontáneamente en tres a ocho semanas y suele pasar desapercibido, pues habitualmente se ubica en el interior de las mucosas ^5^^,^^6^. En la sífilis secundaria (90 % de las mujeres gestantes con sífilis), prevalecen las lesiones dermatológicas generalizadas de predominio maculopapular en forma de diana en palmas y plantas ^5^^-^^7^, las cuales desaparecen en dos a seis semanas y entran en periodo de latencia (silente y asintomática) ^6^. En este estadio de latencia, el objetivo del tratamiento es prevenir las complicaciones y la transmisión vertical ^4^^,^^8^^,^^9^.

Un tercio de los pacientes con sífilis no tratada puede progresar a sífilis terciaria, aproximadamente 3 a 15 años después de la infección inicial ^4^^,^^6^^,^^7^. Debido a las medidas de salud pública y a la lenta progresión de la enfermedad, no es frecuente encontrar el estadio avanzado en mujeres en edad fértil y embarazadas ^6^. El cuadro clínico incluye neurosífilis, enfermedad cardiovascular, lesiones infiltrativas en piel, huesos o vísceras y la clásica goma sifilítica ^5^^,^^7^.

El riesgo de transmisión transplacentaria de la sífilis materna primaria es del 70 % y, en casos de sífilis secundaria, del 90 al 100 % ^4^^,^^6^^,^^7^. Los resultados perinatales adversos se presentan en más de la mitad de las mujeres gestantes con enfermedad activa sin tratamiento, incluidos pérdida gestacional, muerte neonatal en 30 a 50 % de los casos, parto prematuro, retardo del crecimiento fetal grave, bajo peso al nacer y múltiples secuelas posnatales irreversibles, especialmente neurológicas, como retardo mental, hidrocefalia, ceguera y sordera ^4^^,^^6^^,^^7^. Sin tratamiento oportuno, la sífilis puede evolucionar a una enfermedad crónica, con complicaciones potencialmente graves ^2^, convirtiéndose en la segunda causa infecciosa más común de muerte fetal en el mundo, responsable del 50 % de los casos que se presentan anualmente ^8^. Además, constituye un factor de riesgo prevenible de morbilidad y mortalidad infantil ^3^.

Los *Centers for Disease Control and Prevention* (CDC) de Atlanta han reportado un incremento anual de la tasa de sífilis congénita en consonancia con la tendencia de la sífilis primaria y secundaria en la población general y en mujeres en edad reproductiva ^9^. En el 2017, los CDC reportaron en Estados Unidos la tasa más alta de sífilis congénita desde 1997, con 23,3 casos por 100.000 nacidos vivos ^9^.

La Organización Mundial de la Salud (OMS) ha certificado la eliminación de la transmisión vertical en varios países. Sin embargo, se documentó un ascenso en las tasas en la región de las Américas, de 307 casos en el 2012 a 339 casos por 100.000 nacidos vivos en el 2016 ^3^. En ese mismo año, en Colombia se reportó una proporción de sífilis congénita de 111 casos por 100.000 nacidos vivos. Se sabe que el control prenatal es una estrategia efectiva para el control de los casos y, aunque las estadísticas revelan una mayor cobertura del control prenatal, esta sigue estando por debajo de lo esperado en los últimos años ^10^.

En Colombia, se registraron 5.395 casos de sífilis gestacional en el 2018, con una razón de 8,3 por 1.000 nacidos vivos y muertos, siendo la población afrodescendiente la más afectada ^11^. En el Valle del Cauca. se estimó una razón de 12 casos por 1.000 nacidos vivos y muertos, que superó la registrada a nivel nacional ^11^.

En el 2006, se creó y reglamentó en Colombia el Sistema de Vigilancia en Salud Pública (Decreto 3518 de 2006), disposiciones que posteriormente se compilaron en el Decreto 780 del 2016. Desde entonces, se busca fortalecer el proceso de notificación, garantizar la recolección sistemática de la información sobre las condiciones que afectan la salud de la población y definir las acciones para mitigar los riesgos o daños al bienestar de la comunidad a partir de su análisis. Es el caso de la sífilis gestacional, definida como de notificación obligatoria e incluida en el plan de control de enfermedades a nivel nacional, el objetivo primordial es avanzar en el plan de eliminación de la sífilis congénita. Por otra parte, la Resolución 2338 de 2013 establece directrices para facilitar el acceso al diagnóstico de otras infecciones de transmisión sexual (ITS) que pueden estar relacionadas, o presentarse en concomitancia con la sífilis gestacional, con directrices para el entrenamiento en pruebas rápidas de sífilis y HIV, entre otras de este tipo de infecciones.

Previamente, se indicaba el uso de la prueba no treponémica para el diagnóstico de la sífilis en el control prenatal, pero hoy en día se utiliza la prueba treponémica. Desde el 2014, el Ministerio de Salud y Protección Social viene fortaleciendo los aspectos metodológicos y operativos para la mitigación y control de los casos de sífilis congénita, ajustando la definición de caso y la guía de práctica clínica; además, desde el 2018, se implementa la Ruta Integral De Atención en Salud Materno-Perinatal (RIAMP) con el acompañamiento del Instituto Nacional de Salud como ente responsable de la vigilancia ^12^.

Sin embargo, las cifras de sífilis gestacional en Colombia se incrementaron de 1,3 a 6,6 por 1.000 nacidos vivos entre el 2003 y el 2016, indicadores que se encuentran muy lejos de la meta establecida por la OMS, lo que afecta directamente la presentación de la sífilis congénita, pues se esperaba que para el 2021 se encontrara en alrededor de 0,5 casos o menos (incluidos mortinatos) por 1.000 nacidos vivos ^13^.

El panorama en el Valle del Cauca tampoco resulta alentador: según el reporte del análisis de situación integrado de salud de Santiago de Cali (ASIS) del 2017, entre las situaciones de interés en salud pública de mayor letalidad estuvo la sífilis congénita, con una incidencia de 2,9 en ese año en la ciudad ^14^.

Cali representa alrededor del 50 % del total de la población del Valle del Cauca y sus indicadores tienen un impacto importante a nivel departamental, por lo que se hace necesario describir las características sociodemográficas y clínicas, así como la distribución espacial de la sífilis gestacional en la ciudad en el 201, para determinar la distribución, la frecuencia y la magnitud de esta enfermedad de interés en salud pública, y generar estrategias orientadas a minimizar su presencia y sus repercusiones en la madre y el feto.

## Materiales y métodos

Se hizo un estudio observacional de corte transversal y se analizó la información contenida en la base de datos de nacidos vivos del área de estadística de la Secretaría de Salud Municipal de Cali, la cual es reportada por el DANE a partir de los datos ingresados en los certificados de nacimiento en la plataforma del Registro Único de Afiliados - Nacimientos y Defunciones (RUAF-ND). Se analizaron los datos del Sivigila correspondientes a los casos de sífilis gestacional registrados en Cali en la ficha de notificación nacional (código INS: 750, año 2015), teniendo en cuenta la definición de caso de sífilis gestacional del Instituto Nacional de Salud:

“[…] Toda mujer gestante, puérpera o con aborto en los últimos 40 días con o sin signos clínicos sugestivos de sífilis (por ejemplo: úlcera genital, erupción cutánea, placas en palmas y plantas), con prueba treponémica rápida positiva acompañada de una prueba no treponémica reactiva (VDRL, RPR) a cualquier dilución, que no ha recibido tratamiento adecuado para sífilis durante la presente gestación o que tiene una reinfección no tratada […]” ^2^.

Con base en la meta de la OMS de alcanzar y mantener la tasa de sífilis congénita en alrededor de 0,5 casos o menos por 1.000 nacidos vivos, se revisaron y se analizaron los datos para comparar los resultados. Se revisaron los datos contenidos en el registro, y los casos presentados en el periodo de enero a diciembre del 2018 se agruparon por periodos epidemiológicos. La población objetivo estuvo conformada por todos los casos notificados a través de la ficha correspondiente entre las mujeres residentes en Cali; no se requirió muestreo, pues se utilizó toda la información disponible y se excluyeron los casos procedentes de otros municipios y aquellos con más del 10 % de registro incompleto de las variables de interés del estudio.

Se calculó la razón de la sífilis congénita en Cali en el 2018, se describieron las características sociodemográficas de dicha población (edad, comuna, tipo de régimen de salud, etnia y ocupación) y clínicas (estado gestacional de las pacientes en el momento del diagnóstico: embarazo, parto, puerperio o después del aborto), los datos sobre la prueba treponémica y no treponémica (si se practicó o no y la edad gestacional en el momento de la toma de la muestra), así como lo referente al tratamiento (dosis de penicilina recibidas y edad gestacional al iniciar el tratamiento). Los resultados por comuna (en las 22 comunas del municipio de Cali) se registraron mediante la distribución espacial utilizando el programa Qgis 3.0. Los datos faltantes sobre la dirección de residencia y la comuna se completaron con información contenida en otras bases de datos de la Secretaría de Salud Municipal de Cali (SISBEN, RIPS y nacimientos).

Para el procesamiento de los datos, se utilizó el programa R, versión 3.5.3, y para los cuadros y figuras, Excel, versión 2016.

### Consideraciones éticas

El estudio se clasificó como de riesgo menor al mínimo al utilizar una fuente secundaria de datos. El uso de la base de datos fue autorizado por la Secretaría de Salud Municipal de Cali y el estudio fue avalado por el Comité de Ética Médica y Bioética de Investigación de la Universidad Libre - Seccional Cali (carta de aprobación: 19/09/2019), para garantizar el manejo adecuado y ético de la información.

## Resultados

**Figura 1:**
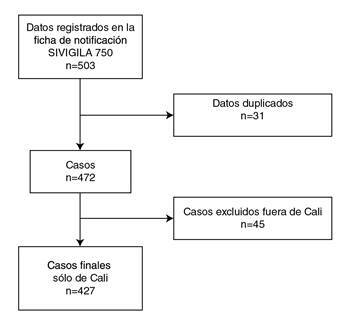
Diagrama de flujo para la selección de los casos de sífilis gestacional en estudio, Cali, 2018

Se revisaron 503 casos de sífilis gestacional registrados en Cali (Colombia), de los cuales se excluyeron 76 que no cumplían los criterios de inclusión (31 que estaban duplicados y otros 45 provenientes de municipios distintos a Cali), para un total de 427 casos definidos como población objeto de estudio (figura 1).

Fuente: Sivigila Cali, 2018, Departamento de Estadística, Secretaría de Salud Municipal de Cali

Se estimó una razón de sífilis congénita de 17 casos por 1.000 nacidos vivos para el 2018 en Cali, calculada a partir de los 427 casos de sífilis gestacional entre los 24.780 nacidos vivos en ese año ^15^. La frecuencia de la sífilis congénita fue mayor en los periodos epidemiológicos 3, 4 y 10, correspondientes a los meses de marzo, abril y septiembre, respectivamente.

En cuanto a la distribución según el régimen de seguridad social en salud, se encontró que la mayoría (57,1 %) de los casos de sífilis gestacional pertenecía al régimen subsidiado, seguido de la población pobre no asegurada (16,6 %), lo que evidenció que el 74 % de los casos diagnosticados se presentaron en población en condición de vulnerabilidad (cuadro 1). Con respecto a la etnia, cerca de 7 de cada 10 casos pertenecían a población mestiza (“otros” según la ficha de Sivigila) y aproximadamente uno de cada cuatro, a poblaciones especiales, destacándose la población afrocolombiana. La ocupación registrada que más predominó (72 %) fue la de ama de casa (cuadro 1). El promedio de edad correspondió a 28 años, con una desviación estándar de ±6,4 años, siendo el grupo de 20 a 34 años el más frecuente (68 %) (cuadro 1).

**Cuadro 1:** Características sociodemográficas de los casos de sífilis gestacional en Cali, 2018

Característica	n	%
Régimen de salud		
Subsidiado	244	57,1
Contributivo	112	26,2
No asegurado	71	16,6
Etnia		
Otros	307	71,9
Negro, mulato, afro,	115	26,9
Indígena	2	0,5
Raizal	2	0,5
Rom, gitano	1	0,2
Ocupación		
Ama de casa	309	72,3
Estudiante	36	8,7
Cesante	13	3,0
Empleado	68	16,0
Edad (años)		
<20	93	22,0
20-34	290	68,0
≥35	44	10,0
Media de edad (años)	DE	Rango
28	± 6,4	15-41
Frecuencia por comuna		
1	6	1,4
2	5	1,2
3	22	5,2
4	12	2,8
5	4	0,9
6	17	4,0
7	25	5,9
8	17	4,0
9	11	2,6
10	8	1,9
11	12	2,8
12	2	0,5
13	47	11,0
14	64	15,0
15	38	8,9
16	22	5,2
17	6	1,4
18	12	2,8
19	9	2,1
20	10	2,3
21	63	14,8
22	4	0,9

DE: desviación estándar

Fuente: Sivigila Cali, 2018, Departamento de Estadística, Secretaría de Salud Municipal de Cali

**Figura 2:**
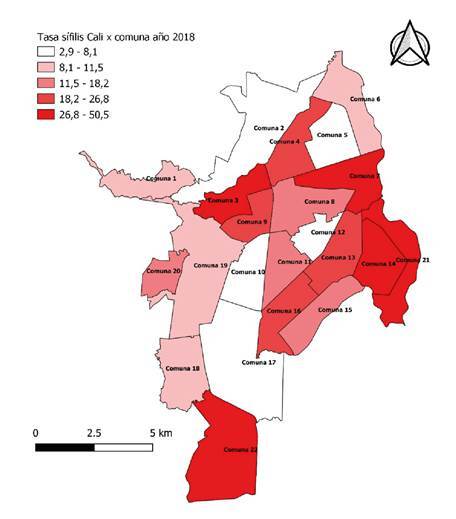
Distribución de casos de sífilis gestacional por comuna en Cali, 2018

En Cali hay 22 comunas, según se observa en el mapa de la figura 2. Al evaluar la distribución espacial de la sífilis gestacional por comuna, se identificó que las tasas más altas se encontraron en las comunas 3, 21, 7, 14, 22, ubicándose por encima de los 26,8 casos por 1.000 nacidos vivos (figura 2). La comuna 3 reportó la tasa más alta, con 50,5 por 1.000 nacidos vivos, seguida de la comuna 21, con 34,5 por 1.000 nacidos vivos, y la comuna 7, con 31,1 por 1.000 nacidos vivos. A estas 3 comunas les siguieron, en orden de frecuencia, las comunas 14, 22 y 13, con una tasa máxima de 28,3 por 1.000 nacidos vivos, y una menor, de 24,1 por 1.000 nacidos vivos

Fuente: Sivigila Cali, 2018, Departamento de Estadística, Secretaría de Salud Municipal de Cali

El 90,4 % de los casos se diagnosticó en el embarazo, el 5,4 %, en el momento del parto, el 2,6 %, en el puerperio, y el 1,6 %, después del aborto (cuadro 2). El 88,5 % de las pacientes tuvo control prenatal y el 54 % lo inició tempranamente (en el primer trimestre). El diagnóstico de sífilis congénita se hace de manera rutinaria con la prueba treponémica y en el estudio se estableció que, en el 8,4 % de los casos, no había registro de esta prueba (cuadro 2).

**Cuadro 2:** Características clínicas de los casos de sífilis gestacional en Cali, 2018

Características	n	%
Diagnóstico en el embarazo	386	90,4
Mujeres gestantes con CPN	378	88,5
Inicio de CPN en el primer trimestre	204	54,0
Mujeres gestantes con prueba treponémica	391	91,6
Mujeres gestantes con prueba no treponémica	412	96,5
Mujeres gestantes que recibieron tratamiento	402	94,1
Pacientes con 1 dosis de penicilina	198	49,3
Pacientes con 2 dosis de penicilina	14	3,5
Pacientes con 3 dosis de penicilina	190	47,2
Contactos de pacientes con sífilis que recibieron tratamiento	246	57,6

CPN: control prenatal

Fuente: Sivigila Cali, 2018, Departamento de Estadística, Secretaría de Salud Municipal de Cali

Se esperaba que el 100 % de las pacientes con prueba treponémica positiva registrara la prueba no treponémica como método de seguimiento. Sin embargo, en el 3,5 % de los casos no se encontró registro de esta. La frecuencia de la prueba no treponémica se reportó por trimestre: 42,5 % en el primero, 37,9 % en el segundo y 19,7 % en el tercero. En cuanto al tratamiento de las pacientes, se determinó que el 5,9 % no recibió tratamiento, como tampoco el 42,4 % de los contactos (cuadro 2).

En el cuadro 3 se discriminan las pacientes con sífilis según su estado en el momento del diagnóstico: embarazo, parto, puerperio, o después de aborto; estas recibieron una dosis de penicilina y fueron en total 198 pacientes. El 86 % de ellas (n=171) se encontraba en estado de embarazo y 16 (8 %) recibieron una dosis de penicilina en el momento del parto. Además, de las 171 mujeres en estado de embarazo, 37 iniciaron el control prenatal entre el primer y segundo trimestres (entre las 12 y 13 semanas). La prueba treponémica fue tardía en 8 de estas 37 mujeres, entre las semanas 17 y 31, cuando se esperaría que se les hiciera en el momento de iniciar su respectivo control prenatal.

**Cuadro 3:** Pacientes con sífilis gestacional que recibieron una dosis de penicilina, Cali, 2018

Dosis de penicilina	Estado de la paciente (n=198)	n	(%)
Pacientes que recibieron una dosis	Embarazo	171	86,4
	Parto	16	8,1
	Puerperio	6	3,0
	Postaborto	5	2,5
Pacientes en embarazo que recibieron una dosis	Embarazo (n=171) (86,4 %)	100	
	Sin dato sobre trimestre de CPN	14	8,1
	I trimestre	83	48,5
	II trimestre	61	35,6
	III trimestre	13	7,6

CPN: control prenatal

Fuente: Sivigila Cali, 2018, Departamento de Estadística, Secretaría de Salud Municipal de Cali

De las 171 mujeres gestantes, 83 iniciaron el control prenatal en el primer trimestre y únicamente recibieron una dosis de penicilina, es decir, el 51,5 % no recibió el tratamiento recomendado. El 57,6 % de los contactos de las gestantes con sífilis gestacional recibieron tratamiento.

## Discusión

La razón de la sífilis gestacional en Colombia en el 2018 fue de 8,3 por 1.000 nacidos vivos ^11^, en tanto que, en Cali, fue de 17 casos por 1.000 nacidos vivos, lo que duplica la cifra nacional. Asimismo, el departamento del Valle del Cauca, junto con Arauca, Buenaventura, Quindío y Chocó, está entre las entidades territoriales con mayor prevalencia de sífilis gestacional (12,9 por 1.000) ^11^.

Se estableció que la mayoría de las pacientes estaba afiliada al sistema de seguridad social, bien fuera al régimen contributivo (26 %) o al subsidiado (57 %), para un total de población asegurada del 83 %, situación similar a la reportada por el Instituto Nacional de Salud, con cifras similares a nivel nacional sobre la presencia de esta enfermedad en las mujeres del régimen subsidiado ^11^. Galeano, *et al.*, reportaron una cifra similar en Cali en el 2010, con el 76,5 % de la población que contaba con afiliación al sistema de salud ^16^. En un estudio de la Universidad de Manizales llevado a cabo en el periodo de 2009 a 2013, se reportó que el 71 % de las pacientes pertenecía al régimen subsidiado, porcentaje semejante al del presente estudio, en tanto que la población en el régimen contributivo fue significativamente menor (15 %) en comparación con la de este estudio, aunque superó el porcentaje de población no asegurada ^17^.

Es evidente que el porcentaje de población asegurada es alto. Como han mencionado Ochoa, *et al.*, un factor determinante del estado de salud de la población es la atención sanitaria, en este caso, de las gestantes, cuyo acceso a los diferentes servicios de salud está a cargo de las entidades administradoras de planes de beneficios (EAPB) ^18^. Sin embargo, estas entidades optan por diferentes modelos de contratación de servicios con las instituciones prestadoras de servicios de salud (IPS), por lo que las actividades de control prenatal resultan fragmentadas y obligan a “las mujeres a desplazarse a diferentes instituciones para acceder al diagnóstico, el tratamiento y la atención especializada” ^18^, con los consecuentes retrasos en la atención y un exceso de trámites que constituye una barrera administrativa ^18^. En el presente análisis, dichas barreras no se abordaron, por lo que se sugiere que se incluyan en futuros estudios.

Según la Organización Panamericana de la Salud (OPS), la tasa de transmisión vertical de sífilis en Brasil aumentó casi el doble entre el 2010 y el 2015, contribuyendo con el 85 % de los casos estimados en la región de las Américas para el 2015 ^19^ . En los países suramericanos, se hace evidente la situación de vulnerabilidad y desigualdad producto de los factores sociales determinantes en salud, por lo que es comprensible que la ocupación prevalente entre las mujeres en el presente estudio haya sido la de ama de casa (72 %), en concordancia con los resultados del estudio de Gramazio, *et al.*, en Guarapuava, estado de Paraná, Brasil, en donde el 75 % de los 40 casos de mujeres con sífilis gestacional tenía dicha ocupación durante el 2014 ^20^. Las mismas similitudes se registraron en cuanto a la etnia, mayoritariamente mestiza en el presente estudio (71,9 %), así como en el de Galeano, *et al.*^16^, y el de Padovani, *et al.,* en el estado de Paraná, Brasil, entre 2011 y 2015 ^21^.

En el presente estudio, se reportó una mayor frecuencia de sífilis gestacional en las mujeres entre los 20 y 34 años, con un 68 % del total de los casos. Al comparar los hallazgos con lo reportado por Gramazio, *et al.*, y Padovani, *et al.*, se encontró un comportamiento similar, con un 75 y un 67 %, respectivamente, de pacientes pertenecientes a este grupo etario en Brasil. En otros estudios, el grupo más afectado fue el de 18 a 24 años ^16^^,^^22^^,^^23^. Una quinta parte de las mujeres del estudio tenía menos de 20 años, lo que las convierte en un grupo de alto riesgo, pues se trata de las adolescentes, quienes tienden a iniciar su vida sexual cada vez más precozmente, sin la conciencia de la necesidad del uso de métodos de protección para prevenir tanto las infecciones de transmisión sexual como los embarazos no deseados ^24^. En otro estudio, Silva, *et al.*, encontraron, además, que las pacientes menores de 18 años presentaban tres veces más riesgo de falla en el tratamiento para la sífilis ^23^, lo que sugiere que la enfermedad es más frecuente en la población joven, pues en estas edades hay mayor exposición.

Al establecer la distribución espacial, se evidenció que las tasas más altas de sífilis gestacional se encontraron en las comunas 3, 21 y 7, ubicadas al oriente de la ciudad, donde reside una población de gran vulnerabilidad socioeconómica, con predominio de los estratos bajos, con un estrato moda de 1 y 3 ^25^, lo cual coincide con los hallazgos de Galeano, *et al.*, quienes reportaron en su estudio que el 93 % de los casos pertenecía a estratos bajos (1 y 2) ^16^. En la comuna 3 se registró la tasa más alta de sífilis gestacional, lo cual podría estar relacionado con su ubicación en el centro de la ciudad, donde predominan las trabajadoras sexuales, el empleo informal y la drogadicción, características sociales que fomentan conductas sexuales de riesgo. Por otra parte, la comuna 22 (estrato moda 6 según Cali en Cifras, 2018) registró una tasa alta (27,5 por 1.000 nacidos vivos) de sífilis gestacional, lo cual concuerda con los hallazgos de Padovani, *et al.*, e indica que, aunque el riesgo de contraerla es mayor en la población vulnerable, cualquiera puede infectarse independientemente de su condición socioeconómica ^21^.

En cuanto a las características clínicas, la mayoría de los diagnósticos se estableció durante el embarazo (control prenatal), al igual que lo registrado por Padovani, *et al.*, en Brasil, con un 78 % de casos diagnosticados durante la gestación, 12 % en el parto o después de un aborto y 9 % en el puerperio ^21^. Por el contrario, en Argentina, Silva, *et al.*, reportaron mayor número de diagnósticos en el puerperio (62,3 %), en tanto que solo el 37,7 % se diagnosticó en el embarazo ^23^.

En cuanto al control prenatal, solo el 88,5 % asistió a estas citas, lo cual evidencia la falta de protección de un porcentaje importante de la población gestante, en coherencia con las cifras de población pobre no asegurada, y constituye un obstáculo en el cumplimiento de la cobertura universal en salud que el país se ha planteado ^26^^,^^27^. En general, lo reportado en la ficha de notificación tanto a nivel local como nacional, indica que el número de mujeres sin control prenatal es considerable, por lo que se requiere una intervención gubernamental inmediata, así este hallazgo no sea generalizable a toda la población gestante. Los hallazgos de Padovani, *et al.*, en Brasil fueron similares, con un 78 % de cobertura de control prenatal ^21^, en tanto que Galeano, *et al.*, encontraron una tasa de cobertura aún menor en Cali (58,2 %) ^16^.

Aunque las pruebas treponémica y no treponémica se practicaron a más del 90 % de las pacientes, lo estipulado es que el 100 % de las pacientes tenga ambas pruebas. Además, las pruebas se hicieron tardíamente, entre el segundo y el tercer trimestres, hallazgo que podría corresponder a la falta de cumplimiento del protocolo de atención, también reportada en Brasil por Gramazio, *et al.*, lo que naturalmente genera subregistro y, en últimas, se convierte en un obstáculo para la vigilancia epidemiológica ^20^. Otra explicación podría ser la inasistencia de las pacientes o un contagio tardío de la infección.

También, hubo un porcentaje de pacientes que no contó con la prueba treponémica pero sí con la no treponémica, lo que podría deberse a una falta de cumplimiento o al desconocimiento del nuevo protocolo por parte de algunos profesionales de la salud, quienes seguirían aplicando guías antiguas sin tener en cuenta los cambios en las directrices para el diagnóstico y el tratamiento.

De cualquier manera, sigue habiendo casos en los que no se detecta tempranamente la infección y, por lo tanto, se retrasa su tratamiento. En la gran mayoría de casos, tampoco se obtuvieron los datos del trimestre en el que se administró el tratamiento a las gestantes, es decir, el diligenciamiento de la ficha de notificación fue incompleto.

Los datos de Colombia en el 2018 revelaron que la mayoría de las pacientes recibió tres dosis de penicilina ^11^; en Cali, el 47,2 % de las pacientes recibió las tres dosis y el 49,3 %, una. Sin embargo, con estos datos no es posible determinar la clasificación del estadio de la sífilis y si el tratamiento fue pertinente, pues se trata de una limitación propia de la ficha de notificación.

En lo que concierne a los contactos, poco más de la mitad recibió tratamiento, lo que concuerda con las cifras del reporte nacional del 2018 ^11^ y refleja una falta de oportunidad en la detección temprana y el tratamiento de la infección, tanto en las mujeres gestantes como en sus contactos. Esto dificulta el control de la enfermedad y refleja una inadecuada aplicación de la ruta integral de atención en salud materno-perinatal. En muchos de los estudios llevados a cabo hasta el momento, se constató el mismo comportamiento: Amador, *et al.*, en Montería, Córdoba, documentaron que el 69 % de los contactos no habían sido tratados, al igual que en Caldas, Manizales (70,2 %), según lo reportado por Agudelo, *et al.*, y en Brasil, por Padovani, *et al.*, (64 %) y Gramazio, *et al.*, (47,5 %) ^17^^,^^20^^-^^22^. Padovani, *et al.*, reportaron los motivos por los cuales los contactos no recibieron tratamiento, entre ellos, la interrupción de la relación de la paciente con su compañero sexual, el resultado negativo de la prueba no treponémica del contacto o la inasistencia a la cita para hacérsela ^21^. En este sentido, como lo mencionan Agudelo, *et al.*, en su estudio en Manizales, el diagnóstico y el tratamiento se ven entorpecidos por las reinfecciones en las gestantes, lo que guarda relación con la “falta de supervisión del tratamiento a la pareja y la falla en la educación en autocuidado de la salud” ^17^. Actualmente, la guía colombiana propone el tratamiento inmediato para los contactos, sin necesidad de prueba, para mejorar el cumplimiento y la oportunidad en el tratamiento. Sin embargo, resultaría pertinente implementar las pruebas no treponémicas de seguimiento para los contactos, con el fin de hacerles un seguimiento más estricto, pues es una población de alto riesgo que queda a la deriva, lo que se interpone al adecuado control y reducción de las tasas de infección. Todo esto nos ubica aún lejos de la meta de 0,5 casos, o menos, por 1.000 nacidos vivos fijada para el 2021.

Los resultados obtenidos por otros investigadores a nivel nacional e internacional y las estadísticas, invitan a reflexionar sobre la creación de estrategias para “invertir en entrenamiento y capacitación en el diagnóstico y el manejo de la sífilis gestacional y la congénita, en el uso adecuado de la penicilina, utilización de las pruebas de diagnóstico rápido y su control de calidad, en el tratamiento de los contactos y en la forma de notificar los casos” ^17^^,^^23^. Es fundamental seguir adecuadamente la ruta materno- perinatal establecida en la guía, y fomentar la integralidad y la oportunidad en la atención de las mujeres gestantes, así como promover el adecuado diligenciamiento de la historia clínica, con el fin de tener un registro de rápido acceso con los antecedentes de las pacientes, especialmente en los casos en que son atendidas en múltiples centros a lo largo de su embarazo, lo cual se presenta con relativa frecuencia.

Por otra parte, se recomienda ajustar la ficha de notificación epidemiológica para establecer en qué fecha exacta se hizo el diagnóstico de la sífilis gestacional (fecha de la prueba treponémica), en qué momento se tomó la prueba no treponémica y en qué fechas se administraron las dosis de penicilina, así como la fecha de inicio del control prenatal, con lo cual se podrían detectar retrasos en la atención que servirían de insumo para futuras intervenciones que corrijan dichas falencias.

Una fortaleza del estudio fue utilizar una base de datos proveniente del ente territorial encargado de la vigilancia epidemiológica de la enfermedad, además de contar con un protocolo nacional e información para comparar con las estadísticas nacionales e internacionales. Entre las limitaciones, están las posibles deficiencias en el diligenciamiento de la información contenida en la ficha, específicamente sobre variables, como el momento de inicio del tratamiento de las pacientes, y datos sociodemográficos, como la dirección y comuna de residencia, lo cual condiciona la calidad de los datos y podría influir en los resultados.
